# Frequent mild head injury promotes trigeminal sensitivity concomitant with microglial proliferation, astrocytosis, and increased neuropeptide levels in the trigeminal pain system

**DOI:** 10.1186/s10194-017-0726-1

**Published:** 2017-02-07

**Authors:** Ashley L. Tyburski, Lan Cheng, Soroush Assari, Kurosh Darvish, Melanie B. Elliott

**Affiliations:** 1 Department of Neurosurgery, Thomas Jefferson University, 1020 Locust Street, Philadelphia, PA 19107 USA; 2 Temple University, Philadelphia, PA USA

**Keywords:** Post-traumatic headache, Migraine, Concussion, Traumatic brain injury, Trigeminal, Microglia, Astrocytosis, Calcitonin gene-related peptide

## Abstract

**Background:**

Frequent mild head injuries or concussion along with the presence of headache may contribute to the persistence of concussion symptoms.

**Methods:**

In this study, the acute ﻿effects of recovery between mild head injuries and the frequency of injuries on a headache behavior, trigeminal allodynia, was assessed using von Frey testing up to one week after injury,﻿ while histopathological changes in the trigeminal pain pathway were evaluated using western blot, ELISA and immunohistochemistry. ﻿

**Results:**

A decreased recovery time combined with an increased mild closed head injury (CHI) frequency results in reduced trigeminal allodynia thresholds compared to controls. The repetitive CHI group with the highest injury frequency showed the greatest reduction in trigeminal thresholds along with greatest increased levels ﻿of calcitonin gene-related peptide (CGRP) in the trigeminal nucleus caudalis. Repetitive CHI resulted in astrogliosis in the central trigeminal system, increased GFAP protein levels in the sensory barrel cortex, and an increased number of microglia cells in the trigeminal nucleus caudalis.

**Conclusions:**

Headache behavior in rats is dependent on the injury frequency and recovery interval between mild head injuries. A worsening of headache behavior after repetitive mild head injuries was concomitant with increases in CGRP levels, the presence of astrocytosis, and microglia proliferation in the central trigeminal pathway. Signaling between neurons and proliferating microglia in the trigeminal pain system may contribute to the initiation of acute headache after concussion or other traumatic brain injuries.

## Background

The incidence of mild traumatic brain injury or concussion worldwide is estimated upwards of 42 million people [1]. Headache, a hallmark symptom of post-concussion syndrome, is frequently the first symptom reported and last one to resolve [2–4]. High incidence rates of post-concussion headache have been reported for athletes (85 to 95.6%) [2, 5–7] and for military service members with concussion (up to 98%) [8, 9]. A substantial subset of US military service members with post-concussion headache reported chronic daily headache [8]. A diagnosis of chronic headache means the patient experiences headache at least 15 days or more per month for three months or more indicating a more severe headache compared to episodic headache [10].

Past head injury, recent injury and the presence of headache may be risk factors for persistent symptoms following concussion that when combined may further exacerbate the outcome from concussion. Studies suggest headache and the presence of migraine were associated with prolonged symptoms following concussion [11, 12]. Conversely, a history of head injury and a shortened time interval between re-injury are considered possible risk factors for persistent symptoms after mild head injury including the transition from acute episodic headache into chronic headache [7, 13–15]. A recent randomized clinical trial of patients with chronic post-traumatic headache demonstrates the interest in studying this disorder; patients with chronic post-traumatic headache unexpectedly failed to show improvement in headache symptoms over time with cognitive behavior therapy intervention, although the refractory nature of the reported headaches may account for this outcome [16].

Models of post-traumatic headache were established by our laboratory using well-known biochemical and sensory behavioral markers in the pain and migraine fields [17, 18]. Previously, our laboratory found cortical injury results in altered trigeminal pain system responses, the presence of meningeal inflammation and trigeminal sensitivity [17–19]. We found that targeting different pathways of excitatory inputs to the trigeminal system can attenuate post-traumatic increases in pain signaling molecules and headache behavior (i.e., trigeminal allodynia) in a murine model of cortical injury [19]. Other laboratories have recently used models of single mild head injury to study mechanisms of altered trigeminal pain signaling acutely after injury expanding on the research and understanding of this understudied problem [20–22]. The purpose of this study was to assess the effects of mild head injuries frequency and recovery time between injuries on trigeminal allodynia, a behavior indicative of headache, in a rat model of mild closed head injury. Our goal was also to characterize several mechanisms proposed to participate in initiating headache behavior following mild closed head injury such as changes in peptidergic responses, astrocytosis, and microglia cellular responses in central regions of the trigeminal pain pathway, the trigeminal nucleus caudalis and/or sensory barrel cortex.

## Methods

### Experimental design

All research involving use of Sprague Dawley rats (Charles River) at approximately 8 weeks of age and weighing 250–300 g was approved and monitored by the Thomas Jefferson University Institutional Animal Care and Use Committee (IACUC) to assure compliance with the provisions of Federal Regulations and the NIH “Guide for the Care and Use of Laboratory Animals”. All animals were housed under a 12-h light/dark cycle in the Thomas Jefferson University, Laboratory Animal Services Facility (AAALAC accredited). All animals underwent a one-week period of acclimation after arriving at the Thomas Jefferson University animal facility before handling.

**Fig. 1:**
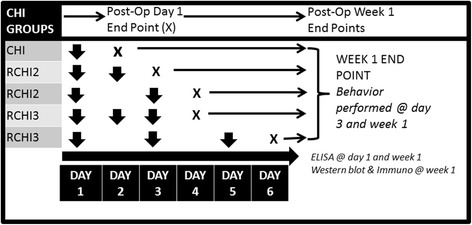
Experimental design showing mild closed head injury groups (CHI) receiving either single (CHI) or repetitive CHI two or three consecutive days (CHI2, CHI3) or with one day of recovery between injuries. All groups underwent baseline behavior testing and post-operative testing for trigeminal allodynia at day 3 and week 1. Tissues were collected at either day 1 or week 1 endpoints for ELISA, western blot, or immunohistochemical analysis

Male Sprague Dawley rats (*n* = 49) were assigned to either single or repetitive mild closed head injury (CHI or RCHI) groups in which RCHI groups receive two or three injuries (RCHI2 or RCHI3), or an incision control group to determine the effects of injury frequency on biochemical and sensory outcomes from injury (Fig. 1). Repetitive CHI groups (RCHI2 and RCHI3) (*n* = 44) were divided into subgroups to receive either injuries on consecutive days (no days of recovery) or every other day (one day of recovery between injuries) to study the effects of recovery time between mild closed head injuries. Pilot data analysis of incision controls undergoing one, two or three incisions showed no significant differences in righting times or observed pain behaviors, *p* = 0.52. Therefore, thirty-six incision controls (*n* = 36) undergoing one incision were run in parallel with experimental groups to study recovery time and frequency of injuries. All animals underwent baseline and post-operative sensory testing at three days and a one week endpoint at which point tissues were collected for either enzyme-linked immunosorbant assay (ELISA), western blot, or immunohistochemical assessments. An additional one day endpoint was also included for ELISA assessment. The effects of single and repetitive CHI on the trigeminal nucleus caudalis (TNC) were examined through immunohistochemistry labeling astrocytes (GFAP) and microglia labeling for ionized calcium binding adapter molecule (Iba-1), and CGRP ELISA. Immunohistochemical and western blot experiments were performed to examine expression of GFAP in the right cortex (ipsilateral to injury). GAPDH protein was used as protein loading control.

### Mild closed head injury

**Fig. 2:**
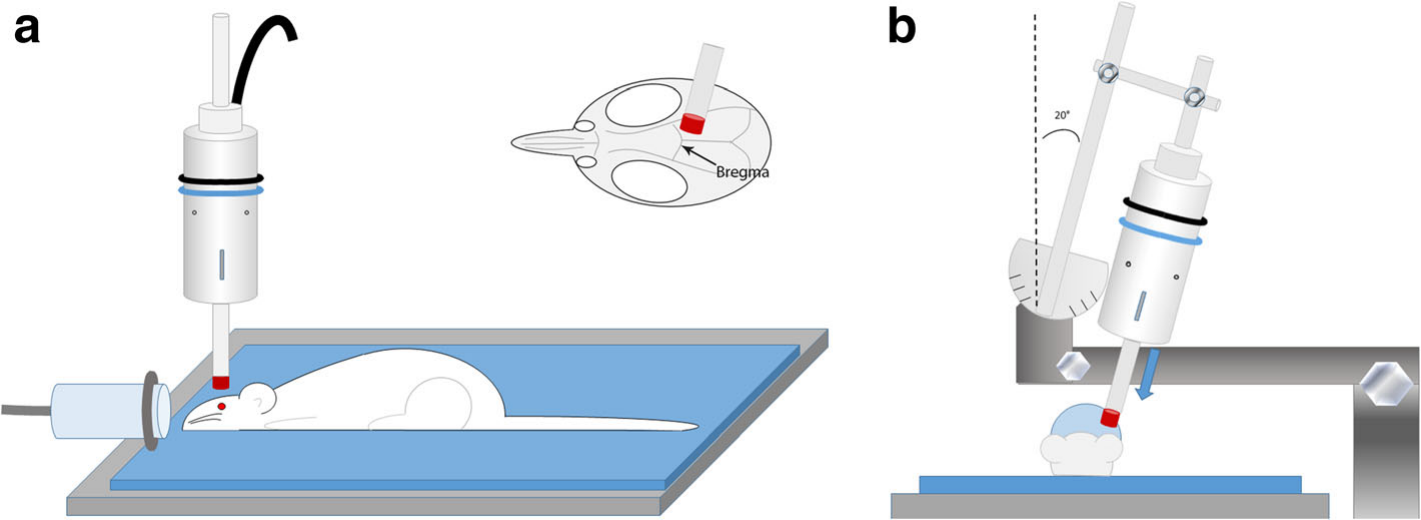
Schematic of mild closed head injury. **a** Cartoon shows foam bedding to support rat while under anesthesia induced through nose cone and impactor with rubber tip. **b** Cartoon positioned at anterior-posterior orientation with nose-cone facing forward shows 20° angle of impactor tip

Mild traumatic brain injury was implemented using a model of mild closed head injury (CHI) adapted from Prins et al. using an electromagnetic device (Impact One, Leica Biosystems, Buffalo Grove, IL) modified with a rubber tip manufactured by the Thomas Jefferson Machine Shop (Fig. 2) [23–25]. The animals were deeply anesthetized using Isoflurane (5% induction and 2–2.5% maintenance). Core temperature was maintained at 37+ 0.5 °C. To ensure aseptic techniques, fur over the surgical site was removed using electric clippers, the area cleaned with alcohol followed by betadine prior to incision. A lidocaine (5–10 μL; 0.5% solution) anesthetic was injected just underneath the scalp at the start of surgery. The body and head of the rat was supported by a soft foam pad ensuring that the head remains in line and level with the body. The foam pad (Foam To Size, Inc., Ashland, VA) cushions the head and body preventing skull fracture while allowing for the head to more freely for a reproducible rotation in the coronal axis. A midline incision was made to expose the skull and the bregma suture, which was used as a landmark for model reproducibility purposes. A modified 10 mm diameter rubber impactor tip was used to deliver the CHI in which the velocity and dwell time were held constant at 5 m/s and 100 milliseconds. The injury displacement was delivered at either a 2.0 or 5.0 mm displacement at a 20° angle from the perpendicular axis to skull surface for all studies (Fig. 2) [23, 24]. There were no fractures to the skull or intracranial bleeding and a 0% mortality rate from this procedure. Immediately prior to impact, the anesthesia nose cone was momentarily removed, and a mild CHI was induced within 5 s after removal from anesthesia. The total time from initiation of anesthesia to removal of the nose cone prior to the zeroing of the impactor tip to the surface of the skull was 10 min. Incision controls rats underwent all procedures except for the impact including the same duration of isoflurane exposure. Once animals regained normal breathing (30–60 s) and prior to fully awakening, they were re-anesthetized with the nose cone (2% Isoflurane) to close the incision with 4.0 silk sutures. A righting response was recorded followed by animals placed under a heat lamp to maintain body temperature throughout recovery.

### Characterization of rat head kinematics

One animal was euthanized and used for determining the CHI model kinematic parameters. Rat skull was exposed and 3 round head pins were attached to the skull (one near the nose and two near the ears) as photo targets. The animal was positioned under the impactor and the head was struck similar to what was described earlier (20° angle and 2 or 5 mm displacement). The positions of the photo targets and their image in a mirror that was placed in front of the animal were recorded using a high speed camera (Phantom, v4.2, Vision Research, Inc., NJ) at 2200 frames per second. The three-dimensional positions of the photo targets were calculated using and in-house code in Matlab and the image processing toolbox (Mathworks, MA). A finite element (FE) model of the rat head with the attached photo targets was developed in LS-Dyna (LSTC, CA). The displacements of the photo targets were given as inputs to the FE model and based on motion of the head center of gravity, the time histories and peak values of the resultant linear and rotational velocities and accelerations of the head were determined. The value of HIC15 (the head injury criterion with 15 ms maximum time interval) was also calculated for the resultant linear acceleration, which is an indication of the intensity of the head acceleration.

### Trigeminal allodynia testing

Trigeminal allodynia testing was performed using von Frey monofilaments (North Coast Medical Inc, CA) with calibrated bending forces (grams) according to the size of the filament. Mechanical allodynia [cutaneous hypersensitivity to a mechanical stimulus that is otherwise innocuous under normal conditions] is a pain response common in patients with migraine, and has also been reported in patients with post-traumatic headache [26–28]. Animals were tested during the morning daylight portion of their circadian cycle (9 am to 12 pm). The periorbital region was selected for allodynia testing as previously described by our laboratory due to this region being innervated by the fifth cranial N, the trigeminal nerve [18, 29]. Von Frey sensory testing is a validated method to test for allodynia in rodents and to evaluate sensory changes in models of inflammatory stimulation [30–33]. For periorbital testing, animals enter the restraint device uncoaxed and without force, tight restraint or change in the degree of restraint for less than 10 min. Periorbital thresholds (grams) were determined by applying the von Frey monofilaments to the periorbital region on the right and left side of the face over the rostral portion of the eye and distal from the suture site for five stimulations. In order for a stimulation to elicit a positive response, the filament must make firm perpendicular contact with the skin causing the filament to bend, and thereby producing a precise bending force as calibrated by the manufacturer. A positive response for periorbital testing was characterized by the following criteria: animal vigorously stroked its face with the forepaw, head withdrawal from the stimulus, or head shaking.

Naïve, baseline periorbital thresholds range for rats is 8–10 g, while a reduction below baseline threshold is considered trigeminal allodynia (≤6 g). While thresholds in some naïve rats may go above 10 g, the upper limit was cutoff at 10 g for all experiments to prevent any cutaneous irritation to the animal. For baseline and post-injury measures, each animal was stimulated five times bilaterally with each filament. A force thresholds (g) was defined as the force at which greater than a 60% (3 out of 5) response frequency for a von Frey stimulus was elicited. Thresholds were presented as the mean threshold (g) ± SEM.

### Enzyme-linked immunoassay (ELISA)

After rats were euthanized with isoflurane and cervical dislocation, specimens of the brain stem tissues rapidly flash frozen in liquid nitrogen were stored at −80 °C. Brainstems were sectioned −14.08 to −17.00 mm to Bregma to include the spinal trigeminal nucleus/TNC. All brainstem samples were homogenized and unpooled in RIPA lysis buffer with EDTA free complete protease inhibitor cocktail tablets using 50 μl/10 mg of tissue (Roche Diagnostics, GMPH, Germany). Homogenates were centrifuged at 14,000 rpm for 15 min at 4 °C. Supernant will be used for follow up studies. Total protein content for each sample was determined using BCA-200 protein assays (Bicin Choninic Acid; Pierce, Rockford, IL). CGRP ELISA Kit (Cayman Chemicals, Ann Arbor, MI, cat# 589001) was used to measure CGRP levels in TNC tissue lysates) according to the manufacturer’s protocol. Each sample was run in duplicate and data were reported as pg/mg of total protein or pg/mL, respectively.

### Western blot analysis

Western blot analyses were performed on right cortex of the brain prepared from incision, sCHI and rCHI rats. Briefly, the tissues were homogenized in radioimmune precipitation assay buffer consisting of 50 mmol/liter Tris–HCl, pH 7.5, 5 mmol/liter EDTA, pH 8, 150 mmol/liter NaCl, 0.1% SDS, 1% Nonidet P-40, and 0.5% sodium deoxycholate. Nuclei and debris were pelleted by centrifugation at 10,000 X rpm for 10 min at 4 °C. The supernatants from spins were collected in fresh tubes to yield lysates. Samples were kept frozen at −80 °C until used. The protein content of each of the tissue preparations was determined using a Bio-Rad BCA protein assay kit (Thermo Scientific, Rockford, IL). Samples were heated for 10 min at 95 °C, and then proteins were routinely resolved on 4–12% NuPAGE Bis-Tris gels in MOPS buffer, or on 4–20% Tris-glycine gels (Invitrogen) at 40 μg/lane. Samples were then transferred onto PVDF membranes. The membranes were then blocked and incubated overnight at 4 °C with mouse Glial fibrillary acidic protein (GFAP) clone GA5 (MAB360, Millipore) overnight at 4 °C. For normalization of signals, blotting was also performed with an anti-GAPDH (anti-glyceraldehyde-3-phosphate dehydrogenase) antibodies (MA5-15738, Thermo fisher; #2118S, cell signaling), followed by incubation with an IRDye 680- or IRDye 800CW-conjugated secondary antibody (Li-Cor). Membranes were imaged with the Odyssey infrared imaging system (Li-Cor), and quantitative densitometric analysis was performed by applying Odyssey version 1.2 infrared imaging software.

### Immunohistochemistry and cell counting

Isoflurane euthanized rats were treated with heparinized saline followed by 4% paraformaldehyde treatment. Brains were post-fixed in 4% paraformaldehyde for 24 h, followed by 24 h in phosphate buffered saline, then transferred to 30% sucrose for storage until sectioning. Frozen sections were cut coronally with a cryostat at – 24 °C (20 μm thickness), and air dried overnight. Cerebrum sections cut −1.0 to −4.0 mm to bregma and TNC sections cut −14.08 to −17.0 mm to bregma were assessed for astrogliois. Immunohistochemical examination of GFAP and was performed for the opthalmic (V1) division of the TNC and the barrel cortex region of the primary sensory (SI) cortex (i.e., barrel cortex; BC) as these regions converge topographically within the trigeminal pain pathway and are corresponding regions to the area testing for trigeminal allodynia. Tissues were incubated in 10% NGS (normal goat serum) in 0.3% Triton-100 for 30 min. Alternate sections were labeled with primary antibodies rabbit GFAP (1:500 Millipore), rabbit Iba-1 (1:500; Wako), overnight at room temperature, and incubated in fluorescent secondary antibodies DyLight 488-conjugated AffiniPure Goat anti-rabbit IgG (Jackson ImmunoResearch) (diluted 1:400 in 10%NGS) for 2 h at room temperature. DAPI (Invitrogen Life Science) mounting media was applied to visualize the nucleus for cell counting Iba-1 microglia cells. Alternate TNC sections were incubated in rabbit anti-β-Amyloid Precursor Protein (1:500; Invitrogen), followed by biotinylated secondary antibody solution (Jackson), VectaStain ABC Reagent for antibody amplification and peroxidase DAB for visualization. Negative control staining was performed by omitting the primary antibodies. All immunohistochemical experiments included an incision control group, experimental group, and a negative control processed at the same time to control for variability with processing. Images were capture using a Nikon Eclipse NI-U microscope with NisElements software (Nikon Instruments, Inc., Melville, NY). When capturing images for an immunohistochemical experiment, exposure times were held constant between specimens.

Stereological methods were used to count cells positive for Iba-1 microglial and DAPI in the TNC by an observer blinded to the experimental conditions. The side ipsilateral to the injury was included in the analysis. Cell counts were measured using a Nikon NIU microscope at a 20× objective for three sampling frames (0.01 μm^2^ each frame) from three sections per animal [34]. When capturing images, exposure times were held constant between specimens and cells with fluorescent staining selected using the thresholding tool from the NisElements software. All section were averaged and reported as the mean ± SE number of positive cells.

### Statistical analysis

A Two-Way ANOVA was used to assess the effects of group and condition (timepoint or recovery time) for trigeminal thresholds. A student’s *T*-test was used to determine side differences for von Frey thresholds. To determine the group effects on righting times and histochemical outcomes, one-way ANOVAs were used at matched time points. ANOVAs were followed by Bonferroni post-hoc tests for multiple comparisons with adjusted *p*-values. All data were analyzed using the GraphPad Prism 5 statistical program. Significance levels were set at *p <* 0.05 for all statistical analyses, adjusted *p*-values are reported, and data are reported as the mean and SEM.

## Results

### Righting responses after injury are dependent on the frequency of head injury in rats

**Fig. 3:**
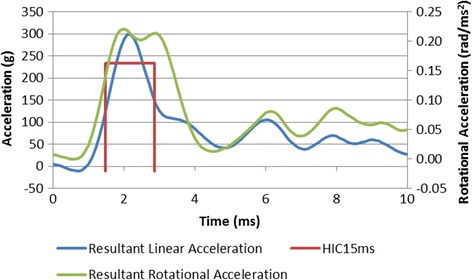
Resultant linear and rotational head accelerations and HIC15 time interval for a representative 5 mm displacement impact. The corresponding HIC15 value is 1157

We found single CHI had similar righting times as the control group. In contrast, RCHI (RCHI2 and RCHI3) resulted in a significantly increased mean righting response time compared to control (Table 1), ANOVA *p* < 0.01. The advantage of using this particular model is that the estimated g force, rotational measures and HIC15 values for our rodent model are sufficient to produce both behaviorial and histopathological changes (gliosis) indicative of concussion (without fracture to the calvarium). In order to evaluate the kinematics parameters, each test was repeated 5 times and the results showed good repeatability. A representative of acceleration data is shown in Fig. 3. The reported values below are average and standard deviation. The impactor velocity was held constant to impact the head at 5 m/s which resulted in movement of the rat’s head with peak linear velocities following impact of 3.0 (0.4) and 0.7 (0.1) m/s for 5 and 2 mm displacements, respectively. For the two displacements, peak linear accelerations (g force) were 284 (22) and 107 (2) g respectively. The corresponding HIC15 values were 1032 (177) and 185 (94) which shows that the severity of the stronger impact was approximately 6 times the milder one. While a number of mechanical and physiological variables exist, a head impact resulting in a HIC15 value over 250 (Mackay, 2007) and peak acceleration over 80 g (Guskiewicz, 2011) is associated with an elevated risk of concussion in humans. The estimated g force and HIC15 values for our rodent model are the sufficient to produce behavior and histological changes (astrocytosis) indicative of mild traumatic brain injury or concussion without fracture to the calvarium. The peak rotational measures for our model were 184 (1) and 105 (5) rad/s for rotational velocity and 243000 (30000) and 211000 (76000) rad/s^2^ for rotational acceleration for 5 and 2 mm displacements respectively. Based on the results of Margulies and Thibault (1992), the rotational thresholds of DAI due to rotational motion increase for a smaller brain mass. The threshold levels for moderate to severe DAI in a baboon with a 145 g brain mass are approximately 260 rads/s and 100000 rad/s^2^. Therefore, for rat brain that is about 2 g, it is expected that for a single CHI the obtained rotational peak values may be much smaller than the threshold of DAI. However, we show that repetitive CHI results in behavioral and histopathological changes in rats indicative of post-concussion headache.

**Table 1:** Righting response times (seconds) for mCHl groups with 5 mm displacement and controls

Groups	Control	mCHl	mCHl2	mCHl3
Mean	122.8	128.4	179.6	157.3
Std. Deviation	24.51	35.95	17.13	26.3
Significance	–	ns	**p<0.01	**p<0.05

###  Trigeminal sensitivity is dependent on the frequency of mild head injury and recovery between injuries

Trigeminal thresholds for all groups were measured using von Frey monofilaments at baseline and following injury as previously described by Macolino et al. [18] in rats with traumatic brain injury. Trigeminal thresholds were reduced bilaterally for the all groups with CHI and RCHI compared to controls *p* < 0.0001 (*F* = 59.16); however, there was no statistically significant difference between the sides (ipsilateral and contralateral to the impact), *p* = 0.11 (*F* = 2.69). Therefore, data was presented as the side with the lowest threshold for all groups (bilateral data not shown).

**Fig. 4:**
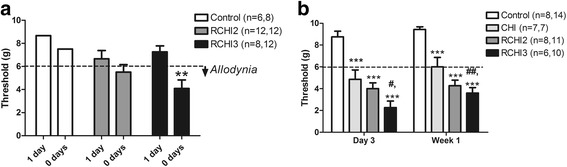
The effect of mild closed head injury (CHI) frequency with or without a day of recovery on trigeminal allodynia thresholds (grams) measured using von Frey filaments. Thresholds for repetitive CHI groups with two or three injuries (RCHI2, RCHI3) and incision controls (control) with 1 day of recovery (1 day) and without a day of recovery (0 days) (**a**). Trigeminal thresholds for RCHI3 (0 days) without a day of recovery between injuries, **p < 0.01 compared to control. Thresholds for CHI, RCHI2, RCHI3 and controls without a day of recovery (0 days) over one week after injury (**b**). Trigeminal thresholds for groups receiving single CHI, RCHI2 and RCHI3, ***p < 0.001 compared to control, ##*p* < 0.01and #*p* < 0.05 compared to CHI. Dashed line at ≤ 6 g represents allodynia

The effects of recovery (zero or one day of recovery) and the frequency of mild head injuries on trigeminal allodynia thresholds were evaluated as these are highly relevant clinical factors of concussion in sports and the military. When the time between injuries was reduced and the frequency of injury was increased, the result was an increase in trigeminal sensitivity (reduced allodynia thresholds). Inducing RCHI on consecutive days without recovery reduced trigeminal thresholds in the group receiving three injuries (RCHI3) compared to controls one week after injury, *p* < 0.01 (*F* = 5.6 group effect) (Fig. 4a). When one day of recovery between injuries for the RCHI3 group was included, there was no significant difference in allodynia thresholds compared to controls (Fig. 4a). All comparisons for recovery effects were made using a 2.0 mm injury displacement. We subsequently, compared trigeminal thresholds for the 2.0 mm and 5.0 mm displacement for CHI groups, and found a the 5.0 mm displacement results in a significant reduction in trigeminal allodynia thresholds, *p* = 0.0062 (data not shown). Therefore, the 5.0 mm injury displacement was used to evaluate the effects of frequency on trigeminal thresholds over one week after injury, as well as for all histopathological experiments on the trigeminal pain system.

Experiments were performed to test the effects of repetitive mild head injury frequency on trigeminal allodynia, where the injury displacement was held constant at 5.0 mm (Fig. 4b). We found an increased frequency of injury reduced the trigeminal thresholds in a graded manner. Single CHI, RCHI2 and RCHI3 showed reduced trigeminal thresholds compared to incision controls, ANOVA *p* < 0.0001 (*F* = 49.66, group effects) and *p* < 0.05 (*F* = 4.6, time effects) (Fig. 4b). Although all injured groups showed trigeminal allodynia compared to the control group, the group with the highest CHI frequency showed the most severe trigeminal sensitivity (largest reduction in trigeminal thresholds). RCHI3 resulted in significantly reduced trigeminal thresholds compared to CHI at day 3 and week 1, *p* < 0.05 and *p* < 0.01.

Repetitive CHI on three consecutive days resulted in the greatest reductions in trigeminal thresholds. Subsequent histopathological analysis evaluated tissues from this group showing the most intense changes in trigeminal sensitivity and were designated as the selected RCHI group.

### Frequent mild head injury induces persistent increases in the pain signaling neuropeptide, CGRP

**Fig 5:**
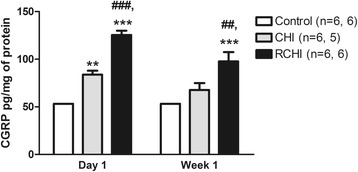
CGRP (pg/mg of protein) measured using an enzyme-linked immunosorbant assay to assess changes in the trigeminal nucleus caudalis at 1 day and 1 week endpoints. The effects of single and repetitive closed head injury (CHI and RCHI) on CGRP levels in the trigeminal nucleus caudalis (*n* = 6 at day 1 and *n* = 5 at week 1) and RCHI groups (*n* = 6/endpoint), **p < 0.01 and ***p < 0.001 compared to incision control (*n* = 6/endpoint), and ##*p* < 0.01 and ###*p* < 0.001 compared to CHI. RCHI was induced on 3 consecutive days

RCHI (on three consecutive days) significantly increased the levels of CGRP in the caudal brainstem compared to incision controls, *p* < 0.01 (*F* = 7.11) (Fig. 5). Increases in CGRP levels after repetitive CHI persist one day to one week endpoints after the last injury, whereas single CHI did not, ANOVA group *p* < 0.0001 (*F* = 46.43) and time *p* < 0.01 (*F* = 8.3). At one day, CHI and RCHI results in increases in CGRP levels compared to incision (ANOVA *p* < 0.0001 and *F* = 62.98), which is consistent with findings for the early time point for trigeminal allodynia in these groups. Notable results show that at one week after the last injury, the RCHI group showed increases in CGRP levels compared to control and CHI, *p* < 0.0026 and *F* = 9.354. In contrast, by one week after injury, the levels of CGRP in the TNC for the single CHI group were not different from control.

### Frequent mild head injury promotes astrocytosis and microglia proliferation in the central trigeminal pain system

**Fig. 6:**
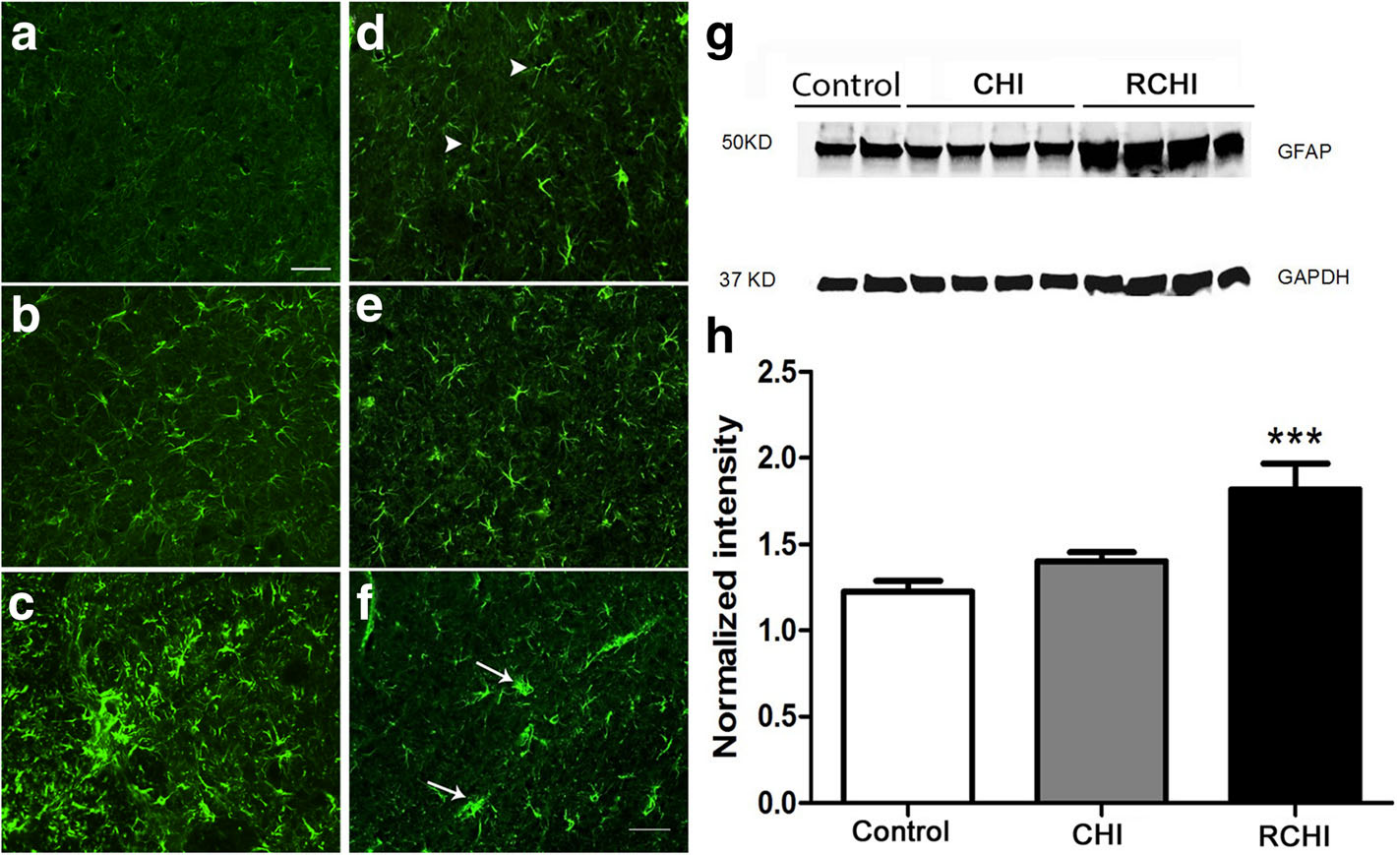
Glial fibrillary acidic protein (GFAP) immunoreactivity in the trigeminal nucleus caudalis (ophthalmic V1 region) and S1 barrel cortex. GFAP staining was performed at week one after incision (**a**), mild closed head injury (CHI) (**b**), or repetitive CHI (RCHI) (**c**). GFAP immunoreactivity in the barrel cortex at week one after control (**d**), CHI (**e**) or RCHI (**f**). GFAP positive astrocytes expressing low levels of GFAP with thin processes in the cortex of controls are indicated by arrow heads (**d**), while reactive astrocytes with increased GFAP immunoreactivity showing thickened or hypertrophied processes in the RCHI cortex are indicated by white arrows (**f**), scale bar = 50 μm. Western blot GFAP expression (**g**) and GFAP expression quantification for the ipsilateral somatosensory cortex of CHI (*n* = 4), and RCHI (*n* = 6) rats compared to control (*n* = 4), *p* < 0.001 (**h**). RCHI was induced on 3 consecutive days

Qualitative immunohistochemical assessment of GFAPimmunoreactivity was performed for the ophthalmic (V1) division of the TNC and the barrel field region of the primary sensory (SI) cortex (i.e., barrel cortex) as these regions converge topographically within the trigeminal pain pathway. Repetitive CHI results in robust GFAP immunoreactivity in TNC tissues compared to controls (Fig. 6a-c). Changes in the pattern of GFAP immunoreactivity and astrocyte phenotype was found in the barrel cortex after RCHI compared to control and CHI (Fig. 6d-f). GFAP positive astrocytes showing thickened processes were present in the TNC and barrel cortex of the repetitive RCHI group, whereas this astrocyte phenotype was absent in the barrel cortex of the CHI or control groups. Primary motor cortex and forelimb regions of the primary sensory cortex were also assessed and did not show differences in GFAP immunoreactivity (data not shown). Western blot analysis demonstrated progressively increased GFAP expression in CHI (14.5%) and RCHI (48.3%) rats on post-operated week one compared with controls, *p* < 0.001 (Fig. 6g-h). These results demonstrate that the injury promotes reactive astrocytes within the trigeminal pain system in both regions including the TNC and sensory barrel cortex.

**Fig. 7:**
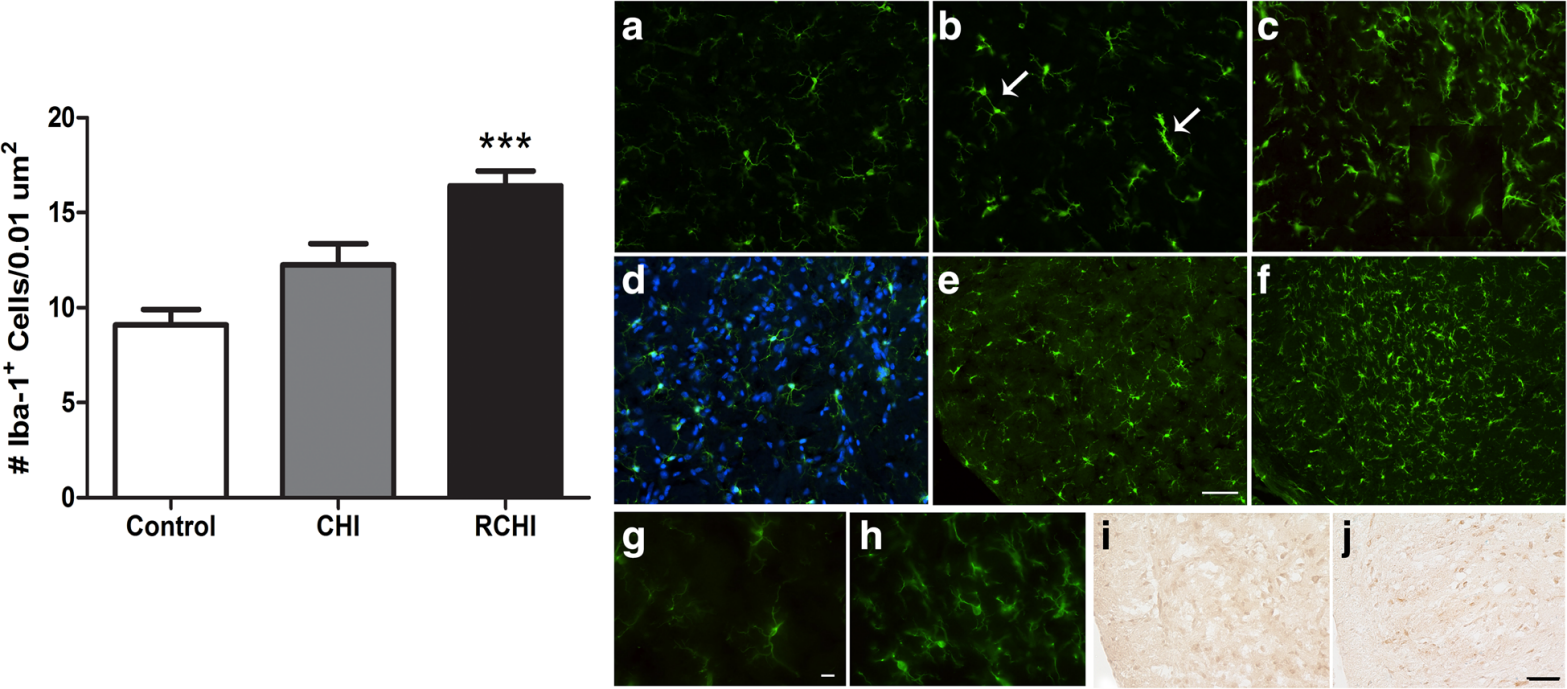
Number of Iba-1 positive cells in the trigeminal nucleus caudalis (TNC) ophthalmic V1 region single and repetitive mild closed head injury (CHI and RCHI), ***p < 0.001 compared to control. Microglial Iba-1 immunoreactivity (**a**-**h**) and β-amyloid precursor protein (**i**, **j**) in the TNC. Iba-1 immunoreactivity for incision control (**a**), CHI (**b**) and RCHI (**c**) rats. Arrows indicate microglia cells coupling or aggregating in the CHI brain, although an increase in the number of microglia cells was not significantly different compared to control during cell counting. Representative images used for cell counting of Iba-1 (*green*) merged with DAPI nuclear stain (*blue*) (**d**). Only cells colabeled with Iba-1 and DAPI were counted. Low power (10×) images and high power (40×) images showing the V1 TNC regions for controls (**c**, **e**, **g**) and RCHI **(f**, **h**). High power images show microglia for a control rat with thin processes (**g**) and Iba-1 positive microglia in the RCHI TNC with thickened, retracted processes and cell proliferation (**h**). β-amyloid precursor protein immunoreactivity is shown for control (**i**) and RCHI (**j**) rat TNC. RCHI was induced on 3 consecutive days. Scale bars **a**-**f** and **i**-**j** = 50 μm, G-H = 10 μm, E-*F* = 100 μm

Quantification of the Iba-1 labeled cells in the ophthalmic (V1) division of the TNC reveals a significant increase in the number of microglia for the RCHI group compared to incision and CHI groups, *p* < 0.001 (Fig. 7). Qualitative immunohistochemical analysis of Iba-1 microglia shows that in addition to the increase in cell number for the RCHI group, thickened or retracted processes were observed (Figure 7c, f, h) compared to the thin processes observed for controls (Figure 7a, e, g) indicating an altered phenotype. These changes in microglia coincide with an absence of axonal injury in the TNC region observed by a lack of accumulated β-amyloid precursor protein.

## Discussion

The more frequent the mild head injury, the worse the trigeminal sensitivity. Results show that the combination of an increased injury frequency with a reduced time between injuries, exacerbates trigeminal sensitivity after mild head injury. The intensification of trigeminal allodynia after frequent mild head injury in a rodent model may be explained by signaling between neurons and proliferating microglia as evidenced by increases in the levels of CGRP and GFAP expression that coincided with increases in the number of microglia cells in the central trigeminal pain pathway.

When the frequency of mild head injury was increased, trigeminal allodynia thresholds were decreased in a graded manner. Data show that the mild head injury group with the most frequent injuries and without recovery time between injuries (injuries on three consecutive days) resulted in the most intense trigeminal sensitivity or greatest reduction in trigeminal allodynia thresholds. We found trigeminal sensitivity was slightly increased earlier after injury for all single and repetitive CHI groups. Our findings are in agreement with acute studies using models of single closed head injury study showing facial thermal allodynia or altered nociceptive processing on a formalin test [22, 35]. Studies have also examined the effects of repetitive mild head injury and time between injuries on cognitive and motor outcomes along with axonal and glial changes in several cortical and subcortical regions [36, 37]. However, this study is the first to demonstrate the effects of repetitive mild head injury frequency and time between injuries on trigeminal mechanical allodynia and corresponding histopathological changes in the central trigeminal pain system.

Our findings show increases in CGRP in the TNC after both single and repetitive CHI, early at one day after injury, which coincides with results for trigeminal allodynia found early for all injured groups. Post-traumatic increases in CGRP and trigeminal sensitivity begin to return to control levels by one week after injury in groups receiving a single CHI, while changes persist in the repetitive CHI groups. Our results are in line with increases in the substance P receptor, NK1 receptor, and thermal allodynia after mild CHI [22]. In a series of previous studies using a focal cortical contusion model, we found increases in several pain signaling molecules, calcitonin gene-related peptide (CGRP), substance P and inducible nitric oxide synthase (iNOS) in the trigeminal pain pathway to correlate with trigeminal allodynia [17, 18, 38, 39]. Substance P and CGRP are excitatory neuropeptides that may be co-released by trigeminal ganglia neurons [40, 41]. The contribution of excitatory mechanisms to pain are appreciated, whereby mechanisms to inhibit neuronal excitation have often been therapeutic targets in pain models [42, 43]. Excitatory input in the trigeminal pain pathway has been studied and blocked acutely in models of traumatic brain injury [17–19, 22, 39]. Notably, CGRP and nitric oxide/NOS are key pain signaling molecules, known to play an important role in migraine pathophysiology and implicated in post-traumatic headache; although substance P has not been shown to play as great a role in migraine headache as CGRP [17, 44–47]. On the other hand, iNOS may serve as an inflammatory signaling molecule that alters the function of neurons or astrocytes. After cortical contusion, iNOS was found to be expressed in part by microglia, as well as neurons in the TNC indicating the NO/NOS pathway remains an important signaling pathway for post-traumatic headache. Inflammatory stimulation of the meninges increased a triggered response of extracellular glutamate in the TNC and trigeminal sensitivity in rats [31, 48]. We propose that persistent inflammatory processes after head injury either from a meningeal and/or periosteal source stimulates an upregulation of CGRP production and release from the trigeminal ganglia neurons synapsing in the TNC [19–21]. Our present findings add to what is currently reported for models of concussion by demonstrating that repetitive mild head injury results in increases CGRP levels in the TNC along with enhanced trigeminal sensitivity that persistent for a week after injury, whereas a single injury does not have the same effect.

Astrocytosis was found in the TNC and in the barrel cortex after repetitive mild closed head injury in rats. Results showing reactive astrocytes after repetitive mild closed head injury are consistent with other studies of repetitive mild traumatic brain injury [49–51]. A study of repetitive closed head injury reported gliosis in the brainstem along with other regions with noted increases when reducing the time between injuries [51]. In contrast to our findings, reactive astrocytes were observed after only a single mild traumatic brain injury using GFAP-luciferase bioluminescence reporter mice indicating the single CHI in the present study may be more mild than that induced by Luo et al. [50]. Luo et al. used a metal tip with the head in a stereotaxic holder, whereas our model used a rubber tip without head restraint allowing for unrestricted movement as has been described by other laboratories [23–25].

Sensory information traveling from periosteal, meningeal, cutaneous or bony tissues of the head potentially undergoing inflammatory processes after concussion follow the same path and have points of convergence within the trigeminal pathway as that of the vibrissae, which are used by rodents to explore their environment. A connection between the whiskers and the trigeminal pain system has been demonstrated behaviorally in mice [52]. Convergence of neurons between the potentially inflamed structures and those not injured or inflamed (ie. whiskers) occurs in the ventral posterior medial thalamus, and ultimately effects may be transmitted to the barrel cortex [53]. Previously, we showed the presence of delayed astrocytosis in the thalamus, an area involved in relaying pain, after a cortical injury in mice [38]. Astrocytosis in the barrel cortex after repetitive mild CHI may indicate either hyper-excitatory mechanisms and/or inflammatory mediators are involved in initiating acute post-traumatic headache. A lack of astrocytosis in other cortical regions (eg. motor cortex and forelimb regions) suggests these changes are specific for the trigeminal circuit; however, it is unclear whether the astrocyte reactivity is due to the afferent drive from potentially hyper-excited neurons, or the mechanical forces of injury. Mechanical loading of astrocyte networks alters their phenotype with implications for brain injury and neuroregeneration [54]. In the TNC, increased CGRP and astrocytosis, combined with a lack of β-APP accumulation suggests afferent-driven excitatory mechanisms in this pain region. Chronic pain models provide support for the role of glutamate regulation in the dorsal horn of the ascending pain pathway [55], and remains an important area to develop for understanding the mechanisms of post-traumatic headache.

We found increases in the number of microglia cells and morphological cell phenotype changes in the central trigeminal pain system (TNC) after repetitive mild closed head injury in the absence of axonal injury in this region. Our results are consistent with studies showing microglia play a role in inflammatory and neuropathic pain [56–59]. Altered microglia function has been implicated in the pathophysiological mechanisms of both migraine and chronic pain conditions [60, 61]. Interestingly, the main contribution to microgliosis in the spinal cord dorsal horn after peripheral nerve injury was found to be from proliferation of resident microglia as opposed to circulating microglial progenitors derived from the bone-marrow [58, 59]. Neuron-microglial signaling including purinergic, fractalkine, and neuroregulin-1 signaling have been implicated in regulating the proliferation of resident microglia in models of peripheral nerve injury [58, 59]. Our results showing an increased number of microglia cells in the TNC after repetitive closed head injury may be from resident microglia in line with those for models of peripheral nerve injury. We base this hypothesis also on the fact that an infiltration of peripheral blood cells would not be expected since this is a mild model of close head injury without tissue loss or necrosis. However, the potential migration of microglia progenitors in response to chemotactic factors cannot be ruled out from this study. We propose that the microglial cell proliferation in the TNC, whether derived from resident microglia or bone-marrow progenitors infiltrating from the blood, plays an essential role in the headache phenotype after mild head injury.

This pre-clinical study of a post-concussion headache model is in line with clinical research that identifies potential risk factors for persistent post-concussion syndrome including frequent head injury, a reduced time interval between injuries, and the presence of headache or migraine [7, 11–15, 62]. Despite the high prevalence of concussions and post-traumatic headache, there is a lack of consensus regarding the management of post-concussion headache [2, 63] including the debate over rest, physical and cognitive, following concussion [64–66]. Our findings are highly relevant when keeping the point in mind that delayed symptoms may surface unexpectedly after head injury in a subset of patients with concussion. A proportion of patients with moderate to severe traumatic brain injury developed post-traumatic headache after the 7-day diagnostic window which is in accordance with the﻿criteria defining post-traumatic headache﻿ by﻿ the International Classification of Headache Disorders (ICHD-3) [9]. The ICHD-3 amends the criteria to include a diagnosis of delayed-onset to be used when the interval between injury and headache onset is greater than seven days [10]. Our data showing acute trigeminal sensitivity at a week after injury, although in line with clinical diagnosis for post-traumatic headache, is considered a study limitation as the persistence of symptoms is an important translational aspect. Moreover, we did not see delayed onset of symptoms in any groups which indicates that our model does account for the multidimensional, psychosocial aspects of pain experienced by humans after head injury, and may also be seen as a study limitation. We acknowledge that the number of injuries and recovery times between injuries were also a study limitation in which an increased frequency of injury or longer recovery times would be expected to alter the outcomes of the study. Our research expands the pre-clinical groundwork into understanding the factors contributing to acute post-traumatic headache, while also accounting for potential mechanisms initiating the headache behavior.

## Conclusions

In conclusion, our results show that an increased frequency and a reduced time interval between injuries worsen headache behavior after mild closed head injury. Repetitive mild closed head injury promotes increases in CGRP levels, astrocytosis and microglial proliferation within the central trigeminal pain system. Findings suggest a role of signaling between neurons and proliferating microglia in the initiation of acute post-concussion headache behavior in rats following repetitive mild closed head injury. Strategies targeting microglial proliferation after post-traumatic headache may provide a therapeutic benefit. Findings demonstrate the impact of injury frequency and recovery time between mild head injuries on altering the trigeminal pain system; factors that are essential to consider when investigating the persistence of post-concussion headache in future pre-clinical and clinical research designs.

## Abbreviations

CGRP: Calcitonin gene-related peptide is a vasoactive neuropeptide involved in pain
CHI: Mild closed head injury model of concussion Closed head injury - model of concussion
GFAP: Glial fibrillary acidic protein is a marker for astrocytes
NOS: Nitric oxide synthase is an enzyme responsible for producing nitric oxide (NO)
TNC: Trigeminal nucleus caudalis is the caudal brain stem region of the trigeminal pain system
